# Lessons from the STOPPCog criteria: prevalence of potentially inappropriate medications for cognition and their association with a cognitive status - a cross-sectional study

**DOI:** 10.3389/fphar.2026.1814603

**Published:** 2026-06-15

**Authors:** Mikołaj Szoszkiewicz, Zofia Szafarkiewicz, Franciszek Szkudelski, Samuel Grabowski, Ewa Deskur-Śmielecka, Agnieszka Neumann-Podczaska, Katarzyna Wieczorowska-Tobis

**Affiliations:** 1 Department of Palliative Medicine, Geriatric Unit, Doctoral School, Poznan University of Medical Sciences, Poznan, Poland; 2 Department of Palliative Medicine, Poznan University of Medical Sciences, The Student Scientific Society of Poznan University of Medical Sciences, Geriatric Scientific Group, Poznan, Poland; 3 Department of Palliative Medicine, Poznan University of Medical Sciences, Geriatric Unit, Poznan, Poland; 4 Senior Institute, VIZJA University, Warsaw, Poland

**Keywords:** antipsychotics, cognitive impairment, dementia treatment, potentially inappropriate medications, STOPPCog criteria

## Abstract

**Introduction:**

Potentially inappropriate medications (PIMs), are common among residents of long-term care institutions (LTCIs) and may contribute to cognitive impairment (CIm). STOPPCog criteria, developed in 2025, support the identification of PIMs relevant to cognition and accuracy of dementia treatment.

**Objectives:**

The study aimed to identify PIMs according to the STOPPCog criteria among residents of long-term care institutions in Poland, and to assess the association between CIm and exposure to specific drug classes.

**Patients and Methods:**

This is a multicenter cross-sectional study in four LTCIs. Medication lists were reviewed using STOPPCog criteria and anticholinergic burden scales. Cognitive status was assessed with the Mini-Mental State Examination (MMSE), and CIm was defined using a conventional cut-off. We compared exposure frequencies between residents with and without CIm and explored factors associated with MMSE using regression models adjusted for key demographic and clinical variables and facility effects.

**Results:**

Of 204 residents, 133 (65.2%) had at least one STOPPCog-listed medication on their medication list. Drugs with anticholinergic burden (ACB) (54.9%), especially one subgroup - antipsychotics (39.2%), were most frequently used within the study sample. Antipsychotics were the only drug class significantly associated with CIm independently of sex, age, comorbidity burden, and facility (p < 0.001). The majority of residents with a previous diagnosis of dementia in their medical records and consequently dementia-specific pharmacotherapy had severe CIm.

**Conclusion:**

There is a high prevalence of PIMs among LTCIs residents. Antipsychotic deprescribing and early diagnosis of CIm should be further evaluated in future prospective studies and clinical practice.

## 1 Introduction

Cognitive impairment (CIm) is best approached as a clinical continuum - from mild cognitive decline to dementia. CIm also encompasses both acute, potentially reversible conditions, such as delirium, and chronic, progressive disorders (Sachdev et al., 2014; McCollum and Karlawish, 2020). A nationwide study revealed that 32% of older citizens of the United States suffered from some degree of CIm (Manly et al., 2022). According to WHO data in 2019, there were about 55.2 million people globally living with dementia, of which 14.1 million patients were from Europe (Albanese et al., 2021). Residents of long-term care institutions are a population with a particularly high risk of CIm. In this group, the prevalence of CIm is significantly higher than in community-dwelling seniors at the same age (Setiyani and Iskandar, 2022; Zhang et al., 2025).

The drug-induced cognitive impairment refers to a decline in cognitive status primarily due to medications (Reimers et al., 2025). Drug-induced cognitive impairment (DICIm) is associated with polypharmacy - a common problem among older patients, with a prevalence of 45% in the age group ≥65 (Delara et al., 2022). DICIm may present with various clinical symptoms and may worsen chronic cognitive disorders, including dementia. At the same time, patients with CIm are at higher risk to develop drug-induced delirium. Reducing polypharmacy and inappropriate medications is a suggested prevention intervention for patients with CIm (Richardson et al., 2020; Fong and Inouye, 2022).

Over the past 2 decades, numerous tools have been developed to identify potentially inappropriate medications; however, no internationally recognized tool specifically addresses cognitive function. The STOPPCog criteria were created by nine experts from Europe–physicians of different specialties and clinical pharmacists. The recommendations underwent two rounds of Delphi validation. The main aim of the STOPPCog criteria was to create a tool that would support evidence-based prescribing decisions and avoid unnecessary DICIm in geriatric patients. STOPPCog consists of six potentially inappropriate medications (PIMs) sections: A- drugs with anticholinergic properties taken daily, B- drugs with sedative properties taken daily, C - drugs that may exacerbate psychotic symptoms in patients with alpha-synuclein pathology, D - drugs used for chronic pain, E − drugs without proven efficacy for dementia taken daily, and F - drugs that are of no proven benefit in severe/advanced stage dementia. Categories A-D concern drugs decreasing cognitive function, whereas Categories E and F relate to the appropriateness of CIm pharmacotherapy (McGettigan et al., 2025).

To date, no empirical study has examined medications affecting cognitive function in the geriatric population using the STOPPCog criteria. Therefore, the present study aimed to examine the prevalence of medications listed in the STOPPCog criteria among long-term care institutions (LTCIs) residents and their association with the cognitive status of this population.

## 2 Materials and methods

### 2.1 Sample and setting

A cross-sectional study was conducted from November 2023 to November 2024 period in four LTCIs in Poland: two in Poznań (a city with a population above 500,000 citizens), one in Pokrzywno (the suburbs of a city with a population above 500,000 citizens), and one in Lisówki (rural area). LTCIs included in this study were residential social care institutions for persons requiring 24-h assistance because of age, disease, or disability and unable to function independently in daily life. DPSs are organized by resident profile and provide accommodation-related, nursing, supportive, and, where relevant, educational services tailored to individual needs.

### 2.2 Recruitment process

**FIGURE 1:**
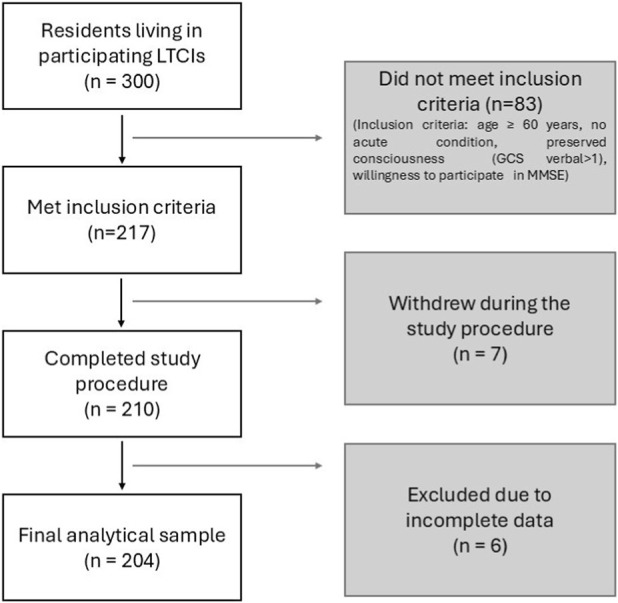
STROBE flow diagram of participant selection and inclusion in the final analysis.

The inclusion criteria were: age at least 60, no acute conditions, consciousness (any verbal response; Glasgow Coma Scale verbal >1), and willingness to participate in the Mini-Mental State Examination. No exclusion criteria were applied except for the absence of medical or pharmaceutical documentation essential for conducting the study. All residents who met the inclusion criteria were assessed by an attending physician. The participant selection process is presented in Figure 1 (see “Results”). A purposive facility-level sampling approach was used to ensure variation in geographic setting. Four LTCIs were included: two located in a large urban area, one in a suburban area, and one in a rural area. Within each selected facility, all eligible residents were assessed, resulting in a final sample of 204 participants.

### 2.3 Data collection process

Data were collected on site by an attending physician through a direct analysis of electronic and paper medical documentation delivered by facility personnel. All clinical data, including the residents’ medical history, were extracted from paper-based records kept on site, with the exception of medication lists, which were retrieved from the computerized record system. As-needed medications were not included in the analysis due to possible recall bias and incomplete documentation. Polypharmacy was defined as the concurrent use of at least five regularly prescribed medications at the time of assessment. The comorbidity burden was assessed with the Charlson Comorbidity Index (CCI) (Charlson et al., 1987). Calculations derived from patients’ medical records. Cognitive status was assessed using the Polish version of the Mini-Mental State Examination (MMSE), administered uniformly by a single physician. For the purpose of this study, CIm was operationally defined as an MMSE score below 24 and further classified into three groups: mild CIm (23–19), moderate CIm (18–11), and severe CIm (0–10) (Tombaugh and McIntyre, 1992). Cognitive impairment identified during the study using the MMSE and cognitive impairment management were compared with cognitive disorder diagnoses documented in participants’ medical records. Documented dementia or mild cognitive impairment (MCI) was considered evidence of a recorded CIm.

### 2.4 Data analysis

All clinical, demographic, functional, and laboratory data were collected and entered into a structured Excel database, designed specifically for the study. Data entry was conducted by one of the researchers and verified by a second investigator for accuracy. All data were collected and analyzed anonymously. The use of artificial intelligence in the creative process was limited solely to linguistic correction using programs such as Deepl, Grammarly and ChatGPT.

The evaluation of PIMs in our cohort was conducted according to the recently developed STOPPCog criteria (McGettigan et al., 2025). PIMs were identified by the same physician who conducted the examination on site, in consultation with two geriatricians and a clinician pharmacist. The prevalence of PIMs was defined as the proportion of patients with at least one potentially inappropriate medication from the STOPPCog list. All STOPPCog sections were applied to our analysis, except the F4 subsection, which concerns deprescribing of medications with no proven benefit in advanced dementia. This recommendation includes essential drug groups such as antihypertensives and antiplatelet agents. The decision regarding continuing or discontinuing the use of these medications should be considered individually, rather than generalized within a cross-sectional framework (Johnell, 2015). Additionally, we have extended the assessment to include several substances not specifically listed in the STOPPCog criteria but commonly prescribed and considered relevant in Polish geriatric practice. These agents have been classified into corresponding STOPPCog groups, based on a consensus of two geriatricians, a clinical pharmacist, and a resident physician in geriatrics. The full list of additionally evaluated medications is presented in Supplementary Table 1. We first evaluated the impact of medications on cognitive status (Sections A–D), using MMSE scores as the reference tool for the presence of CIm. Anticholinergic burden (ACB) was assessed using two standardized scales - the ACB scale and the German ACB scale (Boustani et al., 2008; Kiesel et al., 2018). ACB was calculated as the sum of anticholinergic scores assigned to all regularly prescribed medications included in at least one of these scales. When a medication had different scores across the two scales, the score from the ACB scale was applied. In a separate step, we assessed the adequacy of dementia-specific treatments according to Section E. Each substance potentially received for dementia was specifically assessed by the research team to avoid misclassification (for example, vinpocetine for vertigo).

Participant characteristics and the numbers of PIMs according to the STOPPCog list were summarized using descriptive statistics, reported as counts and percentages. Values were presented as a median with interquartile range (IQR). The chi-square (χ^2^) test was used to assess the prevalence of PIMs in two comparisons: between residents with CIm vs. without CIm and moderate or mild CIm vs. severe CIm. To explore factors associated with a cognitive status, univariable linear regression analyses were performed. Explanatory variables included age, sex, medication count, multimorbidity burden, and PIMs categories from the STOPPCog list with an incidence of at least 5% (antihistamines, benzodiazepines, antipsychotics, Z-drugs, opioids, antiepileptics for chronic pain, and anticholinergics). Variables showing an association at p < 0.1 in univariable analyses were considered for inclusion in multivariable analyses; sex, age, facility (fixed effect), and comorbidity burden were included *a priori* based on a previous literature. Homoscedasticity of residuals was assessed graphically using residual-versus-fitted plots for the multivariable linear regression models, and no evidence of substantial heteroscedasticity was observed. The association between a documented CIm diagnosis in the medical history and MMSE category was examined using a chi-square test with Cramer’s V, and its relationship with cognitive status was further evaluated using a univariable regression model. All analyses were conducted in PQStat (2023; v1.8.6.126, Poland), with statistical significance set at p < 0.05.

### 2.5 Ethical considerations

The study was reviewed by the Bioethics Committee at Poznan University of Medical Sciences (Komisja Bioetyczna przy Uniwersytecie Medycznym im. Karola Marcinkowskiego w Poznaniu), ref. no. KB-836/22, which formally confirmed that the project was a non-experimental study and, under Polish law and GCP regulations, did not require ethics committee approval. The present analysis was based on routine MMSE assessment results and anonymized medical documentation, including medication lists. No identifiable personal data were used. Therefore, no study-specific informed consent or proxy consent procedure was required for this analysis.

## 3 Results

### 3.1 Description of the study sample

All residents who met inclusion criteria were further evaluated (n = 217). During study procedure, seven residents (3.2%) withdrew from participation. Furthermore, six residents (2.8%) were excluded due to incomplete data (Figure 1).

LTCIs, long-term care institutes; GCS, Glasgow Coma Scale; MMSE, Mini-Mental State Examination; The population of our study consisted of 204 participants. The median age of patients was 79 years (IQR 71–88), ranging from 60 to 102 years. The study involved 130 women (63.7%) and 74 men (36.3%). The number of prescribed medications ranged from 1 to 20 per patient, with a median of eight medications (IQR 6–11). Polypharmacy affected 171 (83.8%) patients; among them 79 patients received ten or more medications. 33 (16.2%) participants were taking fewer than five medications at the time of assessment.

A high prevalence of chronic conditions characterized the study population. The median CCI was 5 (IQR 4–6). The conditions present in more than 20% of the study population were: hypertension (72.6%), urinary incontinence (28.3%), previous stroke (26.0%), diabetes mellitus (24.5%), and ischemic heart disease (23.0%). CIm, defined as an MMSE score below 24, occurred in 155 patients (76.0%). Among patients with CIm, 34.8% of the population had mild, 34.8% moderate, and 30.4% severe CIm.

### 3.2 The prevalence of medication potentially impairing cognition

Table 1 presents a detailed list of PIMs defined by the STOPPCog criteria. A total of 133 patients took at least one drug or drug combination included in the STOPPCog criteria A-D Among these patients, 45 (33.8%) received two such medications and 62 (46.6%) received three or more. One patient with severe CIm (MMSE = 4) received seven substances: olanzapine (ATC: N05AH03), tramadol (ATC: N02AX02), quetiapine (ATC: N05AH04), baclofen (ATC: M03BX01), hydroxyzine (ATC: N05BB01), zolpidem (ATC: N05CF02), and carbamazepine (ATC: N03AF01). The most common prescribed medication groups were drugs or drug combinations with an anticholinergic burden score ≥2. One anticholinergic subgroup, antipsychotics, were particularly commonly prescribed. Other medications were prescribed less frequently, with opioids and benzodiazepines being the most commonly used. Quetiapine accounted for the majority of antipsychotics used (Table 1). Nearly two-thirds of patients (n = 37) receiving quetiapine were administered a single evening dose of 25–50 mg.

**TABLE 1:** Potentially inappropriate medications (PIMs) according to STOPPCog criteria. All STOPPCog criteria are listed; within each criterion, individual agents are reported only if they were used by ≥3% of patients.

Criteria	Number of patients exposed	Percentage of population
Drugs with anticholinergic properties taken daily		
Specific antidepressants	**6**	**2.9%**
Bladder antimuscarinics that cross the BBB	**10**	**4.9%**
* Solifenacin (G04BD08)*	7	3.4%
Gastrointestinal antimuscarinics that cross the BBB	**1**	**0.5%**
First generation antihistamines	**15**	**7.4%**
*Hydroxyzine (N05BB01)*	11	5.4%
First- and second-generation antipsychotics	**80**	**39.2%**
* Quetiapine (N05AH04)*	56	27.5%
* Risperidone (N05AX08)*	10	4.9%
* Tiapride (N05AL03)*	9	4.4%
* Olanzapine (N05AH03)*	7	3.4%
Any drug or drug combination with an anticholinergic burden (ACB) score ≥ 2	**112**	**54.9%**
Drugs with sedative properties taken daily		
Benzodiazepines	**19**	**9.3%**
* Estazolam (N05CD04)*	9	4.4%
Z-drug hypnotics	**15**	**7.4%**
* Zolpidem (N05CF02)*	12	5.9%
Drugs that may exacerbate psychotic symptoms in patients with alpha-synuclein pathology		
Anticholinergic drugs for Parkinson’s disease tremor	**1**	**0.5%**
Dopamine receptor agonists	**2**	**1.0%**
Monoamine oxidase type B inhibitors 5	**1**	**0.5%**
Drugs used for chronic pain		
Opioids	**37**	**18.1%**
* Tramadol (N02AX02)*	35	17.2%
Anti-epileptic drugs, including gabapentinoids	**16**	**7.8%**
* Pregabalin (N02BF02)*	14	6.9%
Anti-spasticity drugs that cross the BBB	**5**	**2.5%**

BBB, blood-brain barrier; PIMs, potentially inappropriate medications.

Bold values indicate counts within classes; non-bold values refer to individual preparations.

Table 2 compares residents with and without exposure to at least one PIM according to the STOPPCog list. There were no significant differences between the groups, except for the number of prescribed medications. Residents exposed to PIMs had a median of three more prescribed medications than those without PIM exposure.

**TABLE 2:** Demographic and clinical characteristics of residents with and without exposure to at least one STOPPCog-listed potentially inappropriate medication (PIM).

Variable	PIMs present (n = 133)	No PIMs (n-71)	Test result – p-value
Age (median)	79	78	0.73^a^
Sex, male, n (%)	47 (35.3%)	27 (38.0%)	0.35^b^
Comorbidity burden - CCI index (median)	5	5	0.90^a^
Number of drugs (median)	6	9	**<0.001** ^a^
MMSE score (median)	18	19	0.06^a^

^a^ Mann-Whitney test.

^b^ chi-square.

PIM, potentially inappropiate medications; CCI, index–Charlson Comorbidity Index; MMSE, Mini-Mental State Examination.

Bold values indicate counts within classes; non-bold values refer to individual preparations.

The following tables show the prevalence of medications from the STOPPCog list in relation to the presence of CIm (Table 3) and severe CIm within the subgroup with CIm (Table 4). In the overall sample, antipsychotic use was more frequent among residents with CIm than among those without CIm n = 67 (43.2%) vs. n = 13 (26.5%). Within the CIm subgroup, antipsychotics were also prescribed more frequently in patients with severe CIm compared with those with mild or moderate CIm n = 26 (55.3%) vs. n = 41 (38.0%). No other medication class showed a comparable pattern across both analyses.

**TABLE 3:** Prevalence of drug classes from STOPPCog criteria in relation to presence of CIm in total cohort (n = 204): CIm% by exposure group and chi-square (χ^2^) test p-values.

Exposure/Variable	CIm (n = 155) - exposure	No CIm (n = 49) - exposure	p (χ^2^)
Any drug or drug combination from the STOPPCog list (n1 = 133)	103 (66.5%)	30 (61.2%)	0.50
Drug or drug combination with anticholinergic burden (n1 = 112)	88 (56.8%)	24 (49.0%)	0.34
First- and second-generation antipsychotics (n1 = 80)	67 (43.2%)	13 (26.5%)	**0.04**
First-generation antihistamines (n1 = 15)	9 (5.8%)	6 (12.2%)	0.13
Benzodiazepines (n1 = 19)	16 (10.3%)	3 (6.1%)	0.38
Z-drugs (n1 = 15)	10 (6.5%)	5 (10.2%)	0.38
Opioids (n1 = 37)	30 (19.4%)	7 (14.3%)	0.42
Anti-epileptic drugs, including gabapentinoids, used for chronic pain (n1 = 16)	12 (7.7%)	4 (8.2%)	0.92

Cognitive impairment (CIm).

Bold values indicate counts within classes; non-bold values refer to individual preparations.

**TABLE 4:** Prevalence of drug classes from STOPPCog criteria in relation to presence of severe CIm in CIm cohort (n = 155): CIm% by exposure group and chi-square (χ^2^) test p-values.

Exposure/Variable	Severe CIm (n = 47) - exposure	No severe (mild or moderate) CIm (n = 108) - exposure	p (χ^2^)
Any drug or drug combination from the STOPPCog list (n1 = 103)	35 (74.5%)	68 (63.0%)	0.16
Drug or drug combination with an anticholinergic burden (n1 = 88)	29 (61.7%)	59 (54.6%)	0.41
First- and second-generation antipsychotics (n1 = 67)	26 (55.3%)	41 (38.0%)	**0.045**
First-generation antihistamines (n1 = 9)	2 (4.3%)	7 (6.5%)	0.59
Benzodiazepines (n1 = 16)	3 (6.4%)	13 (12.0%)	0.29
Z-drugs (n1 = 10)	4 (8.5%)	6 (5.6%)	0.49
Opioids (n1 = 30)	8 (17.0%)	22 (20.4%)	0.63
Anti-epileptic drugs, including gabapentinoids, used for chronic pain (n1 = 12)	2 (4.3%)	10 (9.3%)	0.28

Cognitive impairment (CIm).

Bold values indicate counts within classes; non-bold values refer to individual preparations.

### 3.3 Factors associated with cognitive status

An association between age, sex, comorbidity burden, number of drugs, and medication classes from the STOPPCog list and MMSE score was analyzed using univariable linear regression (Table 5). Analyses were performed separately for the whole cohort and the subgroup with CIm. Sex and exposure to any PIMs according to the STOPPCog list were significantly associated with the MMSE score. In analyses of individual medication classes, only antipsychotics were independently associated with worse cognitive function, both in the overall cohort and the subgroup with CIm. Antipsychotic exposure showed the largest β coefficient magnitude (−4.1 for the whole group and −2.6 for CIm subgroup). Nevertheless, interpretation should account for potential confounding by indication, since chi-square analyses demonstrated that antipsychotic use was significantly more frequent in residents with CIm. Comorbidity burden was significant in the overall population, whereas the association disappeared in the Cim subgroup. Factors with p-values <0.1 were considered for evaluation in multiple regression models; sex, age, facility, and comorbidity burden were included *a priori*. To address collinearity, we developed two separate multivariable models and presented them in separate tables: one for exposure to any drug or drug combination from the STOPPCog list (Table 6) and one for antipsychotic exposure (Table 7). The association of any PIMs from STOPPCog list with MMSE was borderline significant in the overall cohort and non-significant in the subgroup with CIm. Importantly, exposure to antipsychotics remained significantly associated with poorer cognitive function after adjustment for age, sex, comorbidity burden, and facility.

**TABLE 5:** Univariable linear regression analyses for factors associated with MMSE score in the total sample (n = 204) and in the subgroup with CIm (n = 155).

Variable	β coefficient (total sample)	95% CI	p (total sample)	β coefficient (CIm subgroup)	95% CI	p (CIm subgroup)
Age (years)	−0.25	−0.36 to −0.14	**<0.001**	−0.23	−0.34 to −0.12	**<0.001**
Sex (male)	5.9	3.61–8.20	**<0.001**	5.71	3.42–8.00	**<0.001**
Comorbidity burden (CCI index)	−0.97	−1.62 to −0.33	**0.003**	−0.50	−1.18 to 0.18	0.14
Number of drugs	−0.01	−0.32 to 0.29	0.92	0.10	−0.20 to 0.40	0.51
Any drug or drug combination from the STOPPCog list	−2.54	−4.98 to −0.11	**0.04**	−2.17	−4.59 to 0.24	0.08
Drug or drug combination with an ACB	−1.58	−3.92 to 0.77	0.19	−0.78	−3.1 to 1.54	0.51
First- and second-generation antipsychotics	−4.05	−6.39 to −1.72	**<0.001**	−2.61	−4.90 to −0.33	**0.03**
First-generation antihistamines	2.34	−2.14 to 6.82	0.30	0.51	−4.41 to 5.43	0.84
Benzodiazepines	−0.79	−4.81 to 3.24	0.70	0.76	−3.02 to 4.54	0.69
Z-drugs	1.41	−3.08 to 5.89	0.54	−0.03	−4.72 to 4.65	0.99
Opioids	0.45	−2.59 to 3.49	0.77	1.70	−1.21 to 4.60	0.25
Anti-epileptic drugs, including gabapentinoids, used for chronic pain	1.00	−3.35 to 5.36	0.65	1.39	−2.91 to 5.69	0.52

CIm, cognitive impairment; CI, confidence intervals; CCI, index–Charlson Comorbidity Index; ACB, anticholinergic burden.

Bold values indicate counts within classes; non-bold values refer to individual preparations.

**TABLE 6:** Multivariable linear regression analyses for factors associated with MMSE score in the total sample (n = 204) and in the subgroup with CIm (n = 155). Age, sex, comorbidity burden and presence of any drug or drug combination from the STOPPCog list were included, adjusted for facility fixed effects (dummy variables; facility A as a reference).

Variable	b (total sample)	95% CI	p (total sample)	b (CIm subgroup)	95% CI	p (CIm subgroup)
Age (years)	−0.08	−0.22 to 0.06	0.24	−0.08	−0.22 to 0.06	0.27
Sex (male)	4.22	1.46 to 6.98	**0.003**	4.15	1.42 to 6.88	**0.003**
Comorbidity burden (CCI)	−0.60	−1.25 to 0.05	0.07	−0.20	−0.86 to 0.47	0.56
Any drug or drug combination from the STOPPCog list	−2.30	−4.60 to 0.00	**0.050**	−1.61	−3.91 to 0.69	0.17
Facility B	0.67	−2.52 to 3.86	0.68	0.68	−2.44 to 3.80	0.67
Facility C	0.81	−2.08 to 3.70	0.58	−0.12	−3.02 to 2.78	0.94
Facility D	−0.73	−4.07 to 2.61	0.67	−2.14	−5.37 to 1.10	0.19

CIm, cognitive impairment; CI, confidence intervals; CCI, Charlson Comorbidity Index.

Bold values indicate counts within classes; non-bold values refer to individual preparations.

**TABLE 7:** Multivariable linear regression analyses for factors associated with MMSE score in the total sample (n = 204) and in the subgroup with CIm (n = 155). Age, sex, comorbidity burden and presence of antipsychotics were included, adjusted for facility fixed effects (dummy variables; facility A as a reference).

Variable	b (total sample)	95% CI	p (total sample)	b (CIm subgroup)	95% CI	p (CIm subgroup)
Age (years)	−0.08	−0.22 to 0.06	0.24	−0.09	−0.22 to 0.05	0.22
Sex (male)	3.87	1.13 to 6.60	**0.006**	3.95	1.24 to 6.65	**0.005**
Comorbidity burden (CCI)	−0.54	−1.18 to 0.10	0.10	−0.18	−0.84 to 0.48	0.59
First- and second-generation antipsychotics	−3.30	−5.55 to −1.06	**0.004**	−2.18	−4.35 to −0.01	**0.049**
Facility B	1.04	−2.13 to 4.21	0.52	0.88	−2.24 to 3.99	0.58
Facility C	1.15	−1.70 to 4.01	0.43	0.32	−2.58 to 3.22	0.83
Facility D	−0.48	−3.79 to 2.83	0.77	−2.05	−5.26 to 1.16	0.21

CIm, cognitive impairment; CI, confidence intervals; CCI, Charlson Comorbidity Index.

Bold values indicate counts within classes; non-bold values refer to individual preparations.

### 3.4 Appropriateness of CIm treatment

**FIGURE 2:**
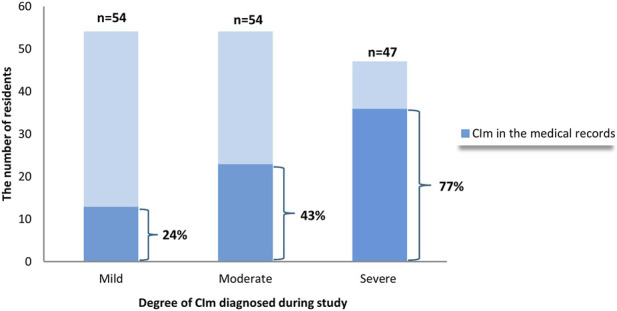
Number of residents with cognitive impairment (CIm) identified during the study and the proportion (%) with a prior CIm diagnosis documented in the medical records. CIm - cognitive impairment.

Among participants with cognitive impairment identified during the study, a prior diagnosis of CIm documented in the medical records (recorded CIm) was present in 72 of 155 individuals (46.5%). The proportion of documented cases increased with cognitive impairment severity, rising from 24.1% in the mild CIm group to 42.6% in the moderate CIm group and 76.6% in the severe CIm group (Figure 2). Two residents with recorded mild CIm passed the MMSE examination with scores above 23 points. The MMSE severity category was significantly associated with the presence of a recorded CIm (Pearson’s chi-square = 28.36, *p* < 0.01), with a moderate-to-large effect size (Cramér’s V = 0.43). In a logistic regression model restricted to residents with CIm, lower MMSE was independently associated with higher odds of having a recorded CIm (OR = 0.89 per one-point increase in MMSE; 95% CI 0.85–0.94; *p* < 0.01). Consequently, the majority of CIm treatment occurred in severe stages. All residents who received acetylcholinesterase inhibitors (donepezil - N06DA02; Rivastigmine -N06DA03) or memantine (N06DX01) had dementia in their medical records. 44 participants took acetylcholinesterase inhibitors or memantine, 13 patients took both medications, and none received antibodies or new-generation drugs. Among 32 patients receiving acetylcholinesterase inhibitors, four had mild CIm, nine had moderate CIm, whereas 19 had severe CIm. Out of 25 memantin users, 16 had severe CIm, eight moderate CIm, and one mild CIm. Daily use of medications without proven efficacy for dementia was observed in the following numbers of residents: 13 vinpocetine (N06BX18), nine piracetam (N06BX03), seven vitamin B, and six other substances (multivitamin supplements, ginkgo bilboa - N06DX02, fish oil, nicergolin - C04AE02).

## 4 Discussion

This study aimed to assess the prevalence of medications included in the STOPPCog criteria among residents of LTCIs and to examine their association with cognitive status in this population. Exposure to any drug or drug combination from the STOPPCog list showed only a borderline association with worse cognitive status in the overall cohort and no significant association in the subgroup with CIm. The most frequent potentially inappropriate substances were drugs or drug combinations with an anticholinergic burden, especially one anticholinergic subgroup - antipsychotics. The prevalence of PIMs was similar in both the CIm group and the cognitively intact group, except for antipsychotic medications, which were used more frequently among those with severe CIm. The use of antipsychotic drugs and age were independent factors associated with worse cognitive status. CIm was underdiagnosed at the mild and moderate stages. Consequently, treatment was provided mostly to residents with severe CIm, where acetylcholinesterase inhibitors and memantine have no proven efficacy.

This study showed a high prevalence of CIm among LTCIs residents - more than 75% of the cohort were affected. By comparison, a study conducted in 12 residential homes in Poland reported a prevalence of 59%; however, that study included a younger population (Kijowska and Szczerbińska, 2018). STOPPCog criteria identify the key medications that may worsen cognitive function; therefore, older adults with CIm should be prioritized for deprescribing. Listed drugs provoke acute cognitive decline and delirium more frequently in patients with CIm (McGettigan et al., 2025; Reimers et al., 2025). A recent analysis reported that, before admission, more than three-quarters of older patients subsequently hospitalized with delirium were exposed to medications that may provoke cognitive decline (Kassie et al., 2019). In our study, 65% of residents received at least one drug or a combination of drugs from the STOPPCog list. Moreover, the prevalence of those medications was similar among residents with and without CIm. The only exception was antipsychotic drugs, which were used more frequently in residents with CIm, particularly at the severe stage. Our findings are concerning, suggesting the need for further assessment of prescribing appropriateness, particularly among residents with CIm.

The most common PIMs identified using the STOPPCog were drugs or drug combinations with an ACB of two or more points. We found that drugs with ACB were present in the majority of our cohort, but were not associated with cognitive decline. The anticholinergic contribution to cognitive decline is well recognized; medications compromise attention and memory by blocking muscarinic receptors of acetylcholine (Hilmer and Gnjidic, 2022). However, clinical evidence linking ACB to CIm is heterogeneous and influenced by the magnitude and duration of exposure, with stronger associations typically observed at higher burden thresholds. A multicenter cross-sectional study showed that only the ACB score of five or more, according to one of the four analyzed scales, was independently associated with CIm among LTCIs residents (Novella et al., 2023). In a recent prospective analysis conducted in ten LTCIs, higher ACB scale scores were associated with 3-year mortality but not CIm (Soraci et al., 2025).

Antipsychotics, one of the anticholinergic subgroups, are high-risk medications in older patients for many reasons; they decrease cognitive status, cause mood changes, but also increase risk of falls and cardiovascular incidents (O’Mahony et al., 2023). The latest Swedish-nationwide study showed that antipsychotics were associated with a higher risk of dementia more than most other anticholinergic drug classes (Zhu et al., 2025). Over the past few years, multiple reports have highlighted the problem of antipsychotic overuse among LTCIs residents and have called for action to counteract the problem (Harrison et al., 2020; Ouslander, 2024). After admission to LTCIs, the prevalence of antipsychotic use typically increases and It is inversely associated with the number of nurses staffing in the facility (Harrison et al., 2020; Travers et al., 2024). Antipsychotics are frequently prescribed for Behavioral and Psychological Symptoms of Dementia (BPSD) in place of non-pharmacological interventions. Facility staff identify BPSD as a major challenge, with antipsychotics being used more frequently when staffing levels are insufficient to provide non-pharmacological support, particularly during nighttime hours (Petersen, 2026).

In our study, 40% of residents received antipsychotic medications—the most commonly prescribed drug class among those listed in the STOPPCog criteria. Antipsychotics were the only drug class administered more frequently in residents with CIm, particularly in those at the severe stage. Prescribing patterns were suggestive of symptomatic use, with quetiapine most frequently administered as a single night-time dose of 25–50 mg. Taken together, these findings indicate that a substantial proportion of antipsychotics were likely used to manage BPSD, such as insomnia and agitation. Antipsychotic use was independently associated with cognitive status in multivariate analyzes after adjustment for sex, age, comorbidity burden, and facility. In the adjusted linear regression model, antipsychotic use was associated with a 3.3-point lower MMSE score in the overall population and a 2.2-point lower MMSE score among residents with CIm. Importantly, the cross-sectional design of this study precludes causal inference, and the observed association between antipsychotic use and cognitive status may be confounded by indication, as these medications were more frequently prescribed to residents with CIm.

Despite the high prevalence of CIm in our cohort, the recorded CIm in medical history referred to 46.5% of them. Moreover, appropriate diagnosis usually occurred in severe CIm. A recent scoping review revealed that underdiagnosis of dementia in residents of LTCIs varies across countries, ranging from 14% to 70%, but is more common in less severe stages (Tewari et al., 2025). A cross-sectional study performed in Austria showed that nearly 70% of the residents without dementia in history showed significant signs of CIm (Auer et al., 2018). The delay in diagnosis appears to be the main factor that contributes to the inadequate CIm treatment in our cohort. Only 9% of residents with mild CIm and 22% with moderate CIm were treated with acetylcholinesterase inhibitors or memantine. Most residents were treated in the severe stage, in which those drugs have no proven benefit (McGettigan et al., 2025). In the end-stage dementia, medication side effects may worsen functional status and economically account for a significant proportion of the patient’s medication expenses (Tjia et al., 2014; Herrmann et al., 2022).

The study has several limitations. Firstly, the sample size was not predetermined. Using purposive facility-level sampling, we recruited participants from four LTCIs in urban and rural settings and controlled for facility-level variation with fixed-effects analyses. Although this strategy yielded a relatively heterogeneous sample, the cohort of 204 participants may still have been underpowered to detect associations involving less prevalent drug classes or medications with lower ACB. The external validity of our findings is limited; therefore, the results should be interpreted with caution and may be most applicable to institutions with similar organizational and resident characteristics. Secondly, cognitive status was assessed using the MMSE, which may be influenced by educational level and sensory impairments (e.g., hearing or visual deficits). Thirdly, the observational design precludes causal associations and excludes clinical interventions. Additional studies with larger samples and a prospective design are needed to examine clinical outcomes.

Our study provides an important complement to the recently published STOPPCog criteria. The prevalence of the assessed drug classes varied, and we identified the most clinically relevant medication-related problems. Medications contributing to ACB constituted the most common PIMs; especially antipsychotics represented the most clinically consequential subgroup. Collectively, these findings suggest that deprescribing priorities in LTCIs residents may differ across medication classes. Reducing antipsychotic use among cognitively impaired residents seems to be a key target for improvement. On the other hand, treatment of the CIm turned out to be rarely adequate, and interventions were implemented at a relatively advanced stage of the disease. This suggests that timely and accurate diagnosis is pivotal for improving CIm treatment.

## 5 Conclusion

In this first worldwide study applying the STOPPCog criteria, we found a high prevalence of PIMs among LTCIs residents. Drugs or drug combinations with an ACB (especially one subgroup–antipsychotics) were most frequently prescribed. Exposure to antipsychotics was independently associated with lower MMSE scores after adjustment for sex, age, comorbidity burden, and facility, underscoring their particular clinical relevance. At the same time, CIm was underdiagnosed at the mild and moderate stages, and treatment was initiated primarily in severe CIm, when clinical benefit is unproven. Taken together, these findings point to a dual gap in LTCIs–high prevalence of anticholinergic medications (especially antipsychotics) among cognitively impaired residents and insufficient early identification and treatment of CIm. There is an urgent need for a large-scale cross-sectional study to evaluate the importance and prevalence of antipsychotic use among LTCI residents. Prospective clinical trials may show the effect of anticholinergic deprescribing and CIm treatment. These studies should be followed by an information campaign addressing the problem of antipsychotic overuse in LTCIs, targeted at physicians and caregivers working in these institutions.

## Data Availability

The raw data supporting the conclusions of this article will be made available by the authors, without undue reservation.
