# Supplementary material for: Lessons from the STOPPCog criteria: prevalence of potentially inappropriate medications for cognition and their association with a cognitive status - a cross-sectional study
Source: Front Pharmacol. 2026 Jun 15;17:1814603. doi: 10.3389/fphar.2026.1814603 (PMC13312536; doi:10.3389/fphar.2026.1814603)
Supplement: Supplementary file 1 [file Table1.docx]

| Supplementary Table 1. List of additional medications evaluated in the study, not explicitly named in the STOPPCog criteria, with corresponding STOPPCog sections. | |
| --- | --- |
| **Active substance** | **STOPPCog section** |
| Hydroxyzine | Section A (A5 – first-generation antihistamines) |
| Tiapride | Section A (A5 – first-generation antipsychotics) |
| Phenothiazine | Section A (A5 – first-generation antipsychotics) |
| Promazine | Section A (A5 – first-generation antipsychotics) |
| Perazine | Section A (A5 – first-generation antipsychotics) |
| Oxazepam | Section B (B2 – benzodiazepine anxiolytics) |
| Bromazepam | Section B (B2 – benzodiazepine anxiolytics) |
| Nicergoline | Section E (E1 – specific nootropics) |
| Vinpocetine | Section E (E1 – specific nootropics) |
